# The outcome of concomitant cholecystectomy with bariatric surgery: a retrospective cohort study

**DOI:** 10.1097/MS9.0000000000000339

**Published:** 2023-03-27

**Authors:** Kadir Yildirim, Ilhan Karabicak, Mahmut F. Gursel, Can Karabicak, Zafer Malazgirt

**Affiliations:** a Department of Surgery, Medical Park Hospital, Samsun; b Department of Public Health, Kastamonu, Turkey

**Keywords:** bariatric surgery, cholecystectomy, cost-effective, gallstones, laparoscopic sleeve gastrectomy, laparoscopic gastric bypass

## Abstract

Gallstones are more common in obese individuals than in healthy individuals. They are diagnosed during the preoperative evaluation for bariatric surgery (BS). However, simultaneous cholecystectomy with BS is still controversial in patients with asymptomatic gallstones in the same session. In this study, the authors aimed to present an analysis of these operations performed with BS in the hospital.

**Materials and Methods:**

The records of 396 patients who underwent BS at Samsun VM Medicalpark Hospital between September 2017 and October 2021 were retrospectively reviewed. The length of hospital stay, operation time, complications, and safety of patients who underwent simultaneous cholecystectomy and BS only were examined.

**Results:**

Of 396 patients, 262 (66.1%) underwent laparoscopic sleeve gastrectomy and 134 (33.8%) underwent laparoscopic gastric bypass surgery. Gallstones were detected during the preoperative examination in 72 (18.1%) of the 396 patients who underwent BS. It was observed that 11 of them had symptoms. No major complications occurred during or after surgery in patients who underwent simultaneous cholecystectomy and only in those who underwent BS.

**Conclusion:**

Simultaneous cholecystectomy with BS does not burden the patient, and complication rates are very low. The procedure is also cost-effective, as patients do not require a second surgery.

## Introduction

Highlights

- Bariatric surgery (BS) is the most effective treatment for obesity.
- Gallstones are more common in obese individuals than in healthy individuals.
- Concurrent cholecystectomy with BS does not burden the patient.
- Concomitant cholecystectomy with BS is cost-effective.

Obesity is a rapidly increasing public health problem worldwide. This problem has serious consequences due to the associated comorbidities. For this reason, numerous studies have been conducted worldwide on treating obesity. It has been shown that the gold standard for the most rapid, durable, and long-term treatment of obesity is bariatric surgery (BS)^1^,^2^.

Gallstones occur in 10–20% of the general population. Gallstones are five times more common in obese people than in healthy people^3^–^5^. It is known that 30–40% of patients undergoing BS develop gallstones due to rapid weight loss. The main reasons are increased cholesterol concentration in bile, decreased cholesterol hypomotility, and increased calcium secretion^6^. There is no consensus on how to treat the asymptomatic gallstones during BS.

Hamad and colleagues and Villegas and colleagues showed that BS without cholecystectomy (CC) has fewer side effects, less surgical time, and a shorter hospital stay. CC after weight loss is advocated as it will facilitate surgery^7^,^8^. However, this approach has significant disadvantages, such as additional cost, morbidity, and hospitalization due to a second surgery. In addition, some patients may develop complications due to gallstones such as pancreatitis, cholecystitis, and cholangitis during the waiting period^9^. Habeeb *et al*.^10^ showed that most of the asymptomatic patients developed symptoms who did not undergo CC during BS and 47% of the asymptomatic patients required surgery within the first year after BS.

In this study, we investigated the early and late outcomes of patients who underwent CC for symptomatic or asymptomatic gallstones during concurrent BS.

## Materials and methods

Between September 2017 and October 2021, a total of 396 patients underwent BS by a single surgeon. The files between these appointments were retrospectively reviewed after approval by the local ethics committee (İstinye University, Protocol No. 22-142). Our work has been reported in line with the STROCSS2021 criteria^11^. Patients with a BMI greater than 40 kg/m^2^ or greater than 35 kg/m^2^ and concomitant diseases (diabetes mellitus, hypertension, obesity-related joint problems, obstructive sleep apnea, etc.) were considered suitable for surgery. Patients who underwent BS for the second time and did not attend the first-year follow-up were excluded from the study. Our research adheres to the principles outlined in the Helsinki Declaration.

Patients who were found to have gallbladder stones on preoperative examination and were previously symptomatic or asymptomatic were included in the study. No BS was performed in patients with chronic liver disease, viral hepatitis, autoimmune hepatitis, drug-induced liver disease, biliary obstruction, chronic kidney disease, coagulopathy, or congestive heart failure.

Age, sex, preoperative BMI, preoperative hepatobiliary ultrasound findings, which BS procedure was performed, duration of surgery, hospital stay, postoperative complications, and first-year BMI were recorded.

All patients underwent laparoscopic sleeve gastrectomy (LSG) or minigastric bypass surgery in the supine position, with endotracheal intubation providing a pneumoperitoneum with a pressure of 18 mm Hg, using five trocars (in the case of CC, an additional 5-mm trocar was inserted from the right subcosta). Gastric resections were performed with a 39-Fr bougie.

### Statistical analysis

Frequency and percentage were reported for categorical variables, and mean and SD were reported for continuous variables. The normality of variables was tested using the Shapiro–Wilk test. The Mann–Whitney *U*-test was used to analyze continuous variables that were not normally distributed. The *χ* ^2^-test was used for the comparison of categorical variables. Analysis was performed using the Statistical Package for the Social Sciences (IBM SPSS, version 22). Results with a *P* value of less than 0.05 were considered significant.

## Results

Between September 2017 and October 2021, 262 (66.1%) of 396 patients undergoing BS underwent LSG, and 134 (33.8%) underwent laparoscopic gastric bypass (LGB). Gallstones were detected during preoperative examination in 72 patients (18.1%) who underwent BS, and all patients underwent simultaneous CC. Of the patients with gallstones, 61 were asymptomatic, while 11 had symptoms such as biliary colic, acute cholecystitis, and acute biliary pancreatitis. Inflammation and severe adhesions were detected in seven asymptomatic patients during surgery. None of the patients experienced complications during CC. No early and late-term complications such as bleeding, bile leakage, or calcular obstructive jaundice were detected after CC during 1-year follow-up.

In the second postoperative week, 500 mg ursodeoxycholic acid (UDCA) were started for all patients who had BS without gallstones. Acute cholecystitis developed within 1 year after BS surgery in three patients who had no stones in the gallbladder before surgery and had emergency surgery.

For the study, patients were divided into two groups. The first group consisted of the patients in whom CC was performed simultaneously with BS surgery. In contrast, the second group consisted of patients on whom only BS surgery was performed.

There were no differences between the two groups regarding cardiac, respiratory, and renal comorbidities and diabetes. The mean age and preoperative BMI were similar in both groups. Additional demographic data are shown in Table 1.

**Table 1:** Demographic data of patients and comparison of groups.

	Group 1 (*n*=72)		Group 2 (*n*=324)		
	LSG+CC	LGB+CC	LSG	LGB	*P* value
Age (years)	34	38	33	36	0.832
Sex					
Male	14	8	92	39	0.714
Female	36	14	120	73	
BMI (kg/m^2^)	45.3	40.3	45.2	40.1	0.784
One year BMI (kg/m^2^)	26.4	25.4	27.4	25.1	0.922
Length of hospital stay (days)	3	3.2	2.9	3.1	0.523
Operative time (min)	66	104	38	75	**<0.001**

Mann–Whitney *U*-test was used for group comparisons.

LGB, laparoscopic gastric bypass; LSG, laparoscopic sleeve gastrectomy.

Significant *P*<0.005 value are in bold.

The average hospital stay for patients undergoing BS alone was 3 days (2–6 days). For patients who underwent concurrent CC, the hospital stay was 3.1 days (3–6 days). While the average operative time for patients undergoing BS was 38 min for LSG, it was 66 min for patients undergoing LSG+CC. For LGB patients, the operative time was 75 min, and for LGB+CC patients, it was 104 min.

The mean operation time was significantly higher in the patients who underwent concurrent CC, about 30 min (*P*<0.001) compared to BS alone. The length of hospital stay was similar in both groups (3.1 and 3 days, *P*=0.53) (Table 1).

While routine infusion therapy for postoperative pain control was administered at the same dose to all patients, no difference in pain control was observed between the two groups. Postoperative blood transfusion in the early phase was performed in four patients in group 1 and three patients in group 2. Postoperative leakage and mortality were not observed in any of the patients.

## Discussion

BS is the most effective treatment for long-term permanent weight loss and elimination of obesity-related comorbidities^12^. The incidence of gallstones is much higher in obese patients compared to the general population. Increased cholesterol secretion rate, larger gallbladder size, and decreased cholecystokinin levels without a proportional increase in bile salts in obese individuals have been suggested as possible causes of gallbladder diseases such as cholelithiasis, cholecystitis, and cholesterolosis^13^,^14^.

Rapid weight loss after BS is also a risk factor for cholelithiasis. It is known that the risk of gallstone formation increases significantly after BS, and the incidence varies from 10 to 38%. This also carries the risk of bile duct complications.

In the past, concomitant CC has been advocated even when there is no stone in the gallbladder to minimize morbidity due to the risk of gallstone formation after BS and complications that may occur in the next process^15^,^16^. Studies contradicting this view have reported that daily administration of 500 mg UDCA is an effective method to prevent gallstone formation after BS. In this study, all patients in group 2 were treated with UDCA 500 mg for 6 months after BS. At 1-year follow-up, gallstones were detected in only three patients (0.9%), and CC was used in these patients. This finding also shows that prophylactic cholecystectomy is unnecessary^17^,^18^.

Nowadays, many studies advocate for or reject concurrent CC in patients with asymptomatic gallstones during BS. In the studies advocating CC, it is emphasized that the procedure does not add morbidity to the patient and that the patient gets rid of a second surgery. Studies that do not endorse CC suggest that concurrent LC can be performed safely but causes some side effects and unfavorable conditions, such as prolonged surgical time, wound infections, gastrointestinal leaks, pneumonia, and a prolonged hospital stay^7^,^8^.

Habeeb *et al*.^10^ reported in their study that there was no difference in complications where the duration of surgery and postoperative hospital stay were longer in 222 patients who underwent concurrent LC.

Aridi and colleagues showed in operations where they performed LSG and simultaneous LC that the operative time increased by an average of 33 min but that there was no significant increase in mortality, hospital stay, and the number of adverse events associated with LC. This study observed a slightly increased risk of bleeding and pneumonia in patients undergoing concurrent CC^19^.

In a study by Wood *et al*.^19^, it was shown that concurrent LC did not cause major complications or mortality and did not affect the length of hospital stay but increased the operative time by an average of 27 min. In our study, similar results were obtained by Wood and colleagues We demonstrated that concurrent LC did not prolong the hospital stay and did not cause complications, except that it increased the operation time by an average of 30 min.

However, there are also opinions advocating that additional port placement is required because access to the gallbladder is difficult due to visceral fat and a large liver, there are technical difficulties due to prolonged surgery, and the risk of complications is increased with CC performed concurrently with BS^14^,^20^,^21^.

The long-term results of asymptomatic gallstones after BS show us the necessity of CC even when the patient is asymptomatic. Sakorafas *et al*.^22^ and Patel *et al*.^23^ reported that less than 5% of asymptomatic gallstones became symptomatic after BS. One of the largest randomized series by Habeeb showed that 55% of the asymptomatic patients required CC after BS^18^. Acute cholecystitis, persistent biliary colic, empyema of the gallbladder, acute biliary pancreatitis, and calcular obstructive jaundice are the most common symptoms for surgery. In that study, 30 and 85% of the patients needed CC 6 and 12 months after BS, respectively. Their CC incidence is higher than most of the studies. Habeeb explains this higher incidence by the large sample size, significant weight loss within 1 year, and consistent follow-up with close observation.

## Conclusion

Concomitant CC during BS prevents patients from exposing themselves to a second surgery in asymptomatic patients in whom CC is needed in high amounts after BS. This study shows that concomitant CC with BS is safe with a very low complication rate and cost-effective in obese patients with asymptomatic gallstones.

## Ethical approval

This study was approved by the Ethical Review Board Committee of İstinye University, Protocol No. 22-142, and all methods were carried out in accordance with relevant guidelines and regulations.

## Consent

This study does not require informed consent because the data used are de-identified.

## Sources of funding

No source of funding.

## Author contribution

K.Y., M.F.G., and C.K.: data collection, statistical analysis, and wrote the paper. Z.M. and I.K.: assisted in the literature search and writing of the paper. K.Y. and İ.K.: writing the paper. D.M.: team leading, conducting the research, and final editing of the paper.

## Conflicts of interest disclosure

No conflict of interest was declared by the authors.

## Guarantor

Kadir Yildirim, Department of Surgery, Medical Park Hospital, Samsun, Turkey. Ilhan Karabicak, Department of Surgery, Medical Park Hospital, Samsun, Turkey.

## Provenance and peer review

Not commissioned, externally peer reviewed.

## References

[1] Sundbom M . Laparoscopic revolution in bariatric surgery. World J Gastroenterol 2014;20:15135–43. 25386062 10.3748/wjg.v20.i41.15135 PMC4223247

[2] Doulamis IP Economopoulos KP . Transumbilical Roux-en-Y gastric bypass in morbidly obese patients: a systematic review. Int J Surg 2015;20:153–7. 26166741 10.1016/j.ijsu.2015.06.077

[3] Festi D Dormi A Capodicasa S . Incidence of gallstone disease in Italy: results from a multicenter, population-based Italian study (the MICOL project). World J Gastroenterol 2008;14:5282–9. 18785280 10.3748/wjg.14.5282 PMC2744058

[4] Dittrick GW Thompson JS Campos D . Gallbladder pathology in morbid obesity. Obes Surg 2005;15:238–42. 15802067 10.1381/0960892053268273

[5] Raziel A Sakran N Szold A . Concomitant cholecystectomy during laparoscopic sleeve gastrectomy. Surg Endosc 2015;29:2789–93. 25480625 10.1007/s00464-014-4010-z

[6] Wudel LJ Jr Wright JK Debelak JP . Prevention of gallstone formation in morbidly obese patients undergoing rapid weight loss: results of a randomized controlled pilot study. J Surg Res 2002;102:50–6. 11792152 10.1006/jsre.2001.6322

[7] Hamad GG Ikramuddin S Gourash WF . Elective cholecystectomy during laparoscopic Roux-en-Y gastric bypass: is it worth the wait? Obes Surg 2003;13:76–81. 12630618 10.1381/096089203321136638

[8] Villegas L Schneider B Provost D . Is routine cholecystectomy required during laparoscopic gastric bypass? Obes Surg 2004;14:206–11. 15018749 10.1381/096089204322857573

[9] Papavramidis S Deligianidis N Papavramidis T . Laparoscopic cholecystectomy after bariatric surgery. Surg Endosc 2003;17:1061–4. 12712384 10.1007/s00464-002-9169-z

[10] Habeeb T Kermansaravi M Giménez ME . Sleeve gastrectomy and cholecystectomy are safe in obese patients with asymptomatic cholelithiasis. A multicenter randomized trial. World J Surg 2022;46:1721–1733. 35397750 10.1007/s00268-022-06557-2 PMC9174306

[11] Mathew G Agha R . For the STROCSS Group. STROCSS 2021: strengthening the reporting of cohort, cross-sectional and case-control studies in surgery. Int J Surg 2021;96:106165. 34774726 10.1016/j.ijsu.2021.106165

[12] Tucker ON Fajnwaks P Szomstein S . Is concomitant cholecystectomy necessary in obese patients undergoing laparoscopic gastric bypass surgery? Surg Endosc 2008;22:2450–4. 18288531 10.1007/s00464-008-9769-3

[13] Moon RC Teixeira AF DuCoin C . Comparison of cholecystectomy cases after Roux-en-Y gastric bypass, sleeve gastrectomy, and gastric banding. Surg Obes Relat Dis 2014;10:64–8. 23932005 10.1016/j.soard.2013.04.019

[14] Dakour Aridi H Sultanem S Abtar H . Management of gallbladder disease after sleeve gastrectomy in a selected Lebanese population. Surg Obes Relat Dis 2016;12:1300–1304. 27178610 10.1016/j.soard.2016.01.029

[15] Liem RK Niloff PH . Prophylactic cholecystectomy with open gastric bypass operation. Obes Surg 2004;14:763–5. 15318978 10.1381/0960892041590746

[16] Nougou A Suter M . Almost routine prophylactic cholecystectomy during laparoscopic gastric bypass is safe. Obes Surg 2008;18:535–9. 18369687 10.1007/s11695-007-9368-8

[17] Uy MC Talingdan-Te MC Espinosa WZ . Ursodeoxycholic acid in the prevention of gallstone formation after bariatric surgery: a meta-analysis. Obes Surg 2008;18:1532–8. 18574646 10.1007/s11695-008-9587-7

[18] Benarroch-Gampel J Lairson DR Boyd CA . Cost-effectiveness analysis of cholecystectomy during Roux-en-Y gastric bypass for morbid obesity. Surgery 2012;152:363–75. 22938897 10.1016/j.surg.2012.06.013 PMC3488959

[19] Wood SG Kumar SB Dewey E . Safety of concomitant cholecystectomy with laparoscopic sleeve gastrectomy and gastric bypass: a MBSAQIP analysis. Surg Obes Relat Dis 2019;15:864–870. 31060907 10.1016/j.soard.2019.03.004

[20] Iglézias Brandão de Oliveira C Adami Chaim E da Silva BB . Impact of rapid weight reduction on risk of cholelithiasis after bariatric surgery. Obes Surg 2003;13:625–8. 12935366 10.1381/096089203322190862

[21] D’Hondt M Sergeant G Deylgat B . Prophylactic cholecystectomy, a mandatory step in morbidly obese patients undergoing laparoscopic Roux-en-Y gastric bypass? J Gastrointest Surg 2011;15:1532–6. 21751078 10.1007/s11605-011-1617-4

[22] Sakorafas GH Milingos D Peros G . Asymptomatic cholelithiasis: is cholecystectomy really needed? A critical reappraisal 15 years after the introduction of laparoscopic cholecystectomy. Dig Dis Sci 2007;52:1313–25. 17390223 10.1007/s10620-006-9107-3

[23] Patel JA Patel NA Piper GL . Perioperative management of cholelithiasis in patients presenting for laparoscopic Roux-en-Y gastric bypass: have we reached a consensus? Am Surg 2009;75:4706. 19545094

